# Detection and Early Referral of Patients With Interstitial Lung Abnormalities: An Expert Survey Initiative

**DOI:** 10.1016/j.chest.2021.06.035

**Published:** 2021-06-29

**Authors:** Gary M. Hunninghake, Jonathan G. Goldin, Michael A. Kadoch, Jonathan A. Kropski, Ivan O. Rosas, Athol U. Wells, Ruchi Yadav, Howard M. Lazarus, Fereidoun G. Abtin, Tamera J. Corte, Joao A. de Andrade, Kerri A. Johannson, Martin R. Kolb, David A. Lynch, Justin M. Oldham, Paolo Spagnolo, Mary E. Strek, Sara Tomassetti, George R. Washko, Eric S. White, ILA Study Group, Fereidoun Abtin, Katerina Antoniou, Timothy Blackwell, Kevin Brown, Jonathan Chung, Tamera Corte, Bruno Crestani, Peter Crossno, Daniel Culver, Joao de Andrade, Anand Deveraj, Kevin Flaherty, Gunnar Gudmundsson, Hiroto Hatabu, Joe Jacob, Kerri Johansson, Jeff Kanne, Ella Kazerooni, Martin Kolb, David Lynch, Toby Maher, Fernando Martinez, Antonio Morais, Steven D. Nathan, Imre Noth, Justin Oldham, Anna Podolanczuk, Venerino Poletti, Claudia Ravaglia, Elizabetta Renzoni, Luca Richeldi, Geoffrey Rubin, Chris Ryerson, Debasis Sahoo, Sara Tomassetti, Paolo Spagnolo, Mary E. Strek, Rob Suh, Nicola Sverzellati, Dominique Valeyre, Simon Walsh, George Washko, Eric S. White

**Affiliations:** a Pulmonary and Critical Care Division, Brigham and Women’s Hospital, Harvard Medical School, Boston, MA; b Center for Pulmonary Functional Imaging, Brigham and Women’s Hospital, Boston, MA; c Department of Radiological Sciences, University of California at Los Angeles, Los Angeles, CA; d Division of Interventional Radiology, University of California at Los Angeles, Los Angeles, CA; e Department of Radiology, University of California at Davis, Davis, CA; f Division of Pulmonary, Critical Care and Sleep Medicine, University of California at Davis, Davis, CA; g Vanderbilt University Medical Center, Nashville, TN; h Pulmonary, Critical Care and Sleep Medicine, Baylor College of Medicine, Houston, TX; i Interstitial Lung Disease Unit, Royal Brompton Hospital, London, England; j Imaging Institute, Cleveland Clinic, Cleveland, OH; k Boehringer Ingelheim Pharmaceuticals, Inc, Ridgefield, CT; l Department of Respiratory Medicine, Royal Prince Alfred Hospital, and University of Sydney, Sydney NSW, Australia; m University of Calgary, Calgary, AB, Canada; n Firestone Institute for Respiratory Health, Research Institute at St. Joseph’s Healthcare, McMaster University, Hamilton, ON, Canada; o Department of Radiology, National Jewish Health, Denver, CO; p Department of Veterans Affairs Northern California, Sacramento, CA; q Respiratory Disease Unit, Department of Cardiac, Thoracic, Vascular Sciences and Public Health, University of Padova and Padova City Hospital, Padova, Italy; r Section of Pulmonary and Critical Care Medicine, University of Chicago, Chicago, IL; s Department of Experimental and Clinical Medicine, Careggi University Hospital, Florence, Italy; t Division of Pulmonary and Critical Care Medicine, University of Michigan, Ann Arbor, MI

**Keywords:** CT, fibrosis, interstitial lung abnormalities, interstitial lung disease, survey

## Abstract

**Background:**

Interstitial lung abnormalities (ILA) may represent undiagnosed early-stage or subclinical interstitial lung disease (ILD). ILA are often observed incidentally in patients who subsequently develop clinically overt ILD. There is limited information on consensus definitions for, and the appropriate evaluation of, ILA. Early recognition of patients with ILD remains challenging, yet critically important. Expert consensus could inform early recognition and referral.

**Research Question:**

Can consensus-based expert recommendations be identified to guide clinicians in the recognition, referral, and follow-up of patients with or at risk of developing early ILDs?

**Study Design and Methods:**

Pulmonologists and radiologists with expertise in ILD participated in two iterative rounds of surveys. The surveys aimed to establish consensus regarding ILA reporting, identification of patients with ILA, and identification of populations that might benefit from screening for ILD. Recommended referral criteria and follow-up processes were also addressed. Threshold for consensus was defined a priori as ≥ 75% agreement or disagreement.

**Results:**

Fifty-five experts were invited and 44 participated; consensus was reached on 39 of 85 questions. The following clinically important statements achieved consensus: honeycombing and traction bronchiectasis or bronchiolectasis indicate potentially progressive ILD; honeycombing detected during lung cancer screening should be reported as potentially significant (eg, with the Lung CT Screening Reporting and Data System “S-modifier” [Lung-RADS; which indicates clinically significant or potentially significant noncancer findings]), recommending referral to a pulmonologist in the radiology report; high-resolution CT imaging and full pulmonary function tests should be ordered if nondependent subpleural reticulation, traction bronchiectasis, honeycombing, centrilobular ground-glass nodules, or patchy ground-glass opacity are observed on CT imaging; patients with honeycombing or traction bronchiectasis should be referred to a pulmonologist irrespective of diffusion capacity values; and patients with systemic sclerosis should be screened with pulmonary function tests for early-stage ILD.

**Interpretation:**

Guidance was established for identifying clinically relevant ILA, subsequent referral, and follow-up. These results lay the foundation for developing practical guidance on managing patients with ILA.

Interstitial lung diseases (ILDs) comprise a large and heterogeneous group of disorders and are frequently associated with poor outcomes and early mortality.^1^ Given the relative rarity and overlap of ILDs with other clinical entities, patients with ILDs are often diagnosed late in their disease course.^2^ It is possible that earlier identification of ILDs and timely initiation of disease-modifying therapies could help improve clinical outcomes of affected patients,^3^ leading to reductions in morbidity and mortality.^4^

Interstitial lung abnormalities (ILA) are defined as abnormalities on chest CT imaging suggestive of an underlying ILD, in those without a prior clinical diagnosis. Data show that ILA may represent an early stage of ILD in some individuals.^4^, ^5^, ^6^, ^7^, ^8^ Research participants with ILA, and patients with ILD, can have overlapping genetic risk factors^9^, ^10^, ^11^, ^12^, ^13^ and similar but often less severe physiological decrements^11^^,^^14^^,^^15^ and histopathologic findings,^16^ as well as a shared risk of adverse longitudinal outcomes.^10^^,^^17^ ILA are identified in 2% to 10% of adults^11^^,^^14^^,^^18^ and are more common than ILDs. ILA are observed in up to 10% of CT scans in lung cancer screening programs,^19^ and they confer variable risk based on imaging pattern.^20^

Although these findings illustrate the potential clinical importance of identifying ILA, they are not recorded routinely in radiology reports, even at academic centers.^21^ A recent position paper from the Fleischner Society has proposed standardized definitions of ILA and recommended evaluations for incidentally identified ILA.^22^ However, there has been limited information on expert consensus regarding reporting, referral, and follow-up procedures among pulmonologists and radiologists, who are frequently required to make decisions for people found to have ILA on routine clinical scans.

The objectives, therefore, of the current study were to establish expert-based consensus on ILA reporting by radiologists, the imaging findings of ILA that constitute true ILD, and the most appropriate referral and follow-up procedures for individuals with ILA. Finally, we sought consensus on the role of screening in specific populations at high risk of developing ILD.

## Materials and Methods

### Steering Committee and Expert Panel

A Steering Committee (SC) of nine respiratory physicians and thoracic radiologists (one from the United Kingdom and eight from the United States) with experience in ILD was convened and met initially in March 2019.

SC members proposed suitable individuals to comprise an expert panel whose opinions would be surveyed. Each expert panel member was required: (1) to be a currently practicing clinician trained in pulmonology or thoracic radiology; (2) to be willing to participate in two to three surveys (30 minutes each); (3) to have ≥ 5 years of clinical experience in diagnosing, treating, or imaging lung disease; and (4) to have ≥ 3 years of clinical experience with diagnosing and treating ILDs.

Following discussions at the March 2019 meeting, invitees to the expert panel were confirmed. Individuals were invited by personal email; no financial payments were offered to expert panel members. Members of the SC did not participate in answering the survey questions.

### Surveys: Development and Circulation

An anonymized iterative survey process was used to identify key topics of interest regarding early management of ILA. Variations of such an approach have been widely used to systematically seek consensus of expert opinion in multiple fields, including respiratory medicine.^23^, ^24^, ^25^

Objectives of the process were to identify areas of consensus or disagreement on: (1) ILA reporting by radiologists; (2) identification of patients at risk for early stages of ILD; (3) referral criteria and process, and duration of follow-up; and (4) populations that might benefit from screening.

**Figure 1:**
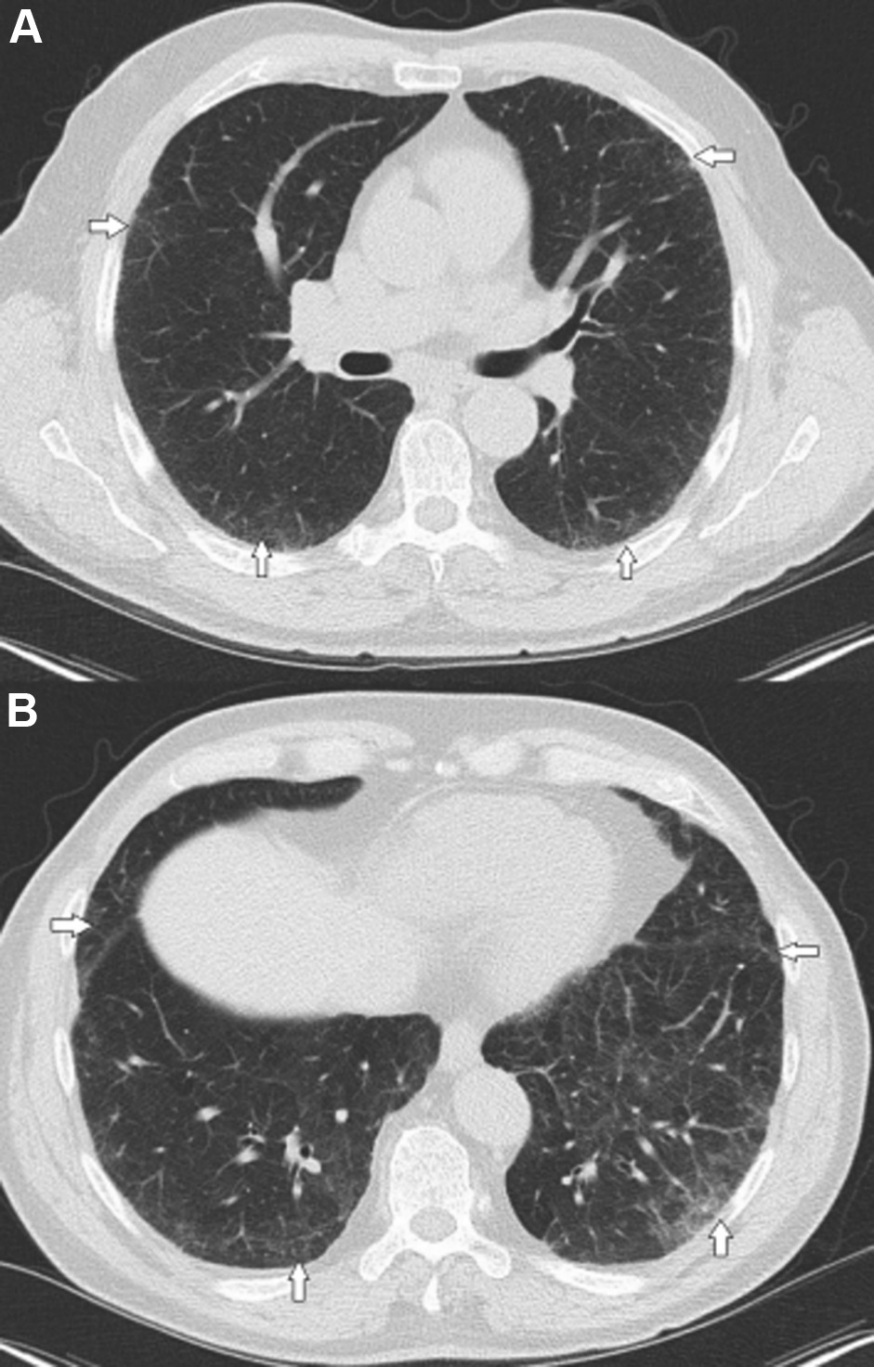
An example of axial images from the carina (A) and lung base (B) from a patient identified with interstitial lung abnormalities in the context of a chest CT scan ordered for routine cancer surveillance. White arrows highlight regions of subpleural reticulation. This chest CT scan is consistent with an indeterminate usual interstitial pneumonia pattern. The patient eventually developed progressive pulmonary fibrosis based on lung function over a 2-year period and was ultimately started on antifibrotic therapy.

An example of ILA identified in a clinical setting is included in Figure 1. At the initial face-to-face meeting, with subsequent editorial finalization, the SC developed approximately 40 questions for inclusion in the first-round questionnaire, to survey the opinions of the expert panel members. The experts were e-mailed a link to the questions by using online survey software. Survey questions were e-mailed, and responses collated, by a third party uninvolved in assessing outcomes; SC members received pooled responses and were blinded to the identity of individual respondents.

Survey One comprised four sections: (1) perceptions of significance of ILA; (2) diagnosis and reporting of incidental ILA; (3) diagnostic testing in asymptomatic patients with incidental ILA; and (4) screening for early ILD in high-risk populations.

Survey One results were reviewed by the SC in a virtual meeting. On the basis of these discussions, the SC developed a revised set of questions for Survey Two. Questions included in Survey Two were based on Survey One responses: they may have had refined wording or response options to probe topics in further detail or to clarify the original intentions of the initial questions, or may have been new questions integrating or addressing earlier categorical or free-text responses. Survey One results were seen and discussed by the SC only and not by the expert panel of respondents. Follow-up questions in the second survey pertained mainly to initial responses in the first survey regarding diagnosis and reporting of incidental ILA, as well as screening for early ILD in high-risk populations. Some questions that achieved consensus in Survey One were not followed up further. Survey Two results were distributed in August 2019.

Surveys One and Two comprised various question types, mainly multiple choice. Some questions were asked contingent on earlier responses. Clinical questions from both surveys and their response options are provided in e-Tables 1 and 2.

When both survey results were available, the SC reviewed the outcomes in a second virtual meeting and identified key conclusions; the SC determined that a third survey was not required. The threshold level for consensus to an individual question was defined a priori as 75% (ie, agreement by ≥ 33 of 44 respondents). The process was completed by September 2019.

## Results

### Expert Panel Characteristics

Of 42 pulmonologists and 13 thoracic radiologists with ILD expertise invited to participate, 32 and 12, respectively, accepted the invitation, comprised the expert panel, and completed Survey One. Among these 44 ILD experts, practicing across 10 countries (Table 1), 84% had > 10 years’ experience in clinical medicine, 95% practiced at an academic center, and 80% saw > 100 patients with ILD yearly. Forty-two members (30 pulmonologists and 12 thoracic radiologists) completed the second survey (Table 1). Of these 42 members, 79% had > 10 years’ experience in clinical medicine, 95% practiced at an academic center, and 77% saw > 100 patients with ILD yearly.

**Table 1:** Steering Committee and Expert Panel Demographic Characteristics

Panel	Steering Committee	Expert Panel (Survey One)	Expert Panel (Survey Two)
No. of participants, n	9	44	42
Female sex, n (%)	1 (11)	9 (20)	9 (21)
Country of practice, n			
Australia	0	1	1
Canada	0	3	3
Denmark	0	1	1
France	0	2	1
Greece	0	1	1
Iceland	0	1	1
Italy	0	4	3
Portugal	0	1	1
United Kingdom	1	6	6
United States	8	24	24
Pulmonologists/thoracic radiologists, n/n	5/4	32/12	30/12

### Survey Results

The 39 questions that achieved consensus agreement in Survey One and Survey Two are presented in Table 2. A full listing of all clinical questions and answers (other than for free-text responses) from both surveys is presented in e-Tables 1 and 2.

**Table 2:** Questions With Answers for Which Consensus (≥ 75% Agreement Among Expert Panel Members) Was Achieved, From Survey 1 and Survey 2

Question	Response That Achieved Consensus	Agreement	No. of Respondents, n
Survey 1			
Perceptions of ILA			
In a person without a clinical diagnosis of ILD, ILA are generally defined by the presence of chest CT imaging features that suggest an underlying ILD: agree or disagree?	Agree	84%	44
Undiagnosed research participants with ILA have demonstrated similar, but often less severe, physiological decrements than those noted on patients with clinically apparent ILD: agree or disagree?	Agree	80%	44
Undiagnosed research participants with ILA have exhibited an increased rate of mortality: agree or disagree?	Agree	77%	44
Diagnosis and reporting of incidentally detected ILA			
In your practice, do you consider honeycombing (irrespective of its extent or distribution) to always indicate the presence of ILD?	Yes	75%	44
[If yes or unsure] Should radiologists include a Lung-RADS “S-modifier” regarding this finding in their report of lung cancer screening CT scans?	Yes	86%	35
[If yes or unsure] Should the conclusion of the radiology report also recommend consideration of a referral to a pulmonologist?	Yes	76%	33
In your practice, do you consider honeycombing (irrespective of its extent or distribution) to always indicate the presence of a fibrosing ILD?	Yes	75%	44
[If yes or unsure] Should radiologists include a Lung-RADS “S-modifier” regarding this finding in their report of lung cancer screening CT scans?	Yes	86%	36
[If yes or unsure] Should the conclusion of the radiology report also recommend consideration of a referral to a pulmonologist?	Yes	78%	36
In your practice, do you consider nondependent subpleural reticulation occupying ≥ 5% of the lung scan (without honeycombing or traction bronchiectasis) to always indicate the presence of fibrosing ILD?			
[If yes or unsure] Should radiologists include a Lung-RADS “S-modifier” regarding this finding in their report of lung cancer screening CT scans?	Yes	88%	16
[If yes or unsure] Should the conclusion of the radiology report also recommend consideration of a referral to a pulmonologist?	Yes	87%	15
In your practice, do you consider centrilobular ground-glass nodules or patchy ground-glass opacity (without honeycombing, traction bronchiectasis, or significant subpleural reticulation) to always indicate the presence of ILD?	No	93%	44
In your practice, do you consider centrilobular ground-glass nodules, or patchy ground-glass opacity (without honeycombing, traction bronchiectasis or significant subpleural reticulation) to always indicate the presence of fibrosing ILD?	No	95%	44
Testing of referred asymptomatic patients			
An asymptomatic patient is referred to clinic based on the presence of either honeycombing or traction bronchiectasis on a CT scan. If not already done, should HRCT imaging be ordered?	Yes	84%	44
An asymptomatic patient is referred to clinic based on the presence of either honeycombing or traction bronchiectasis on an HRCT scan. Which of the following tests, if any, would you order in your practice?	Full pulmonary function tests (spirometry plus lung volumes and measurement of diffusion capacity)	95%	44
An asymptomatic patient is referred to clinic based on the presence of subpleural reticulation occupying ≥ 5% of the lung scan (without honeycombing or traction bronchiectasis). If not already done, should HRCT imaging be ordered?	Yes	89%	44
An asymptomatic patient is referred to clinic based on the presence of subpleural reticulation occupying ≥ 5% of the lung scan (without honeycombing or traction bronchiectasis) on HRCT imaging.	Full pulmonary function tests (spirometry plus lung volumes and measurement of diffusion capacity)	98%	44
An asymptomatic patient is referred to clinic based on the presence of centrilobular ground-glass nodules or patchy ground-glass opacity (without honeycombing, traction bronchiectasis, or significant subpleural reticulation). If not already done, should HRCT imaging be ordered?	Yes	75%	44
An asymptomatic patient is referred to clinic based on the presence of centrilobular ground-glass nodules or patchy ground-glass opacity (without honeycombing, traction bronchiectasis, or significant subpleural reticulation) on HRCT imaging. Which of the following tests, if any, would you order in your practice?	Full pulmonary function tests (spirometry plus lung volumes and measurement of diffusion capacity)	75%	44
Interpreting pulmonology tests			
An asymptomatic patient with either honeycombing or traction bronchiectasis undergoes pulmonary function testing. What reduction from baseline in FVC do you consider clinically significant warranting a referral to a pulmonologist with expertise in ILD?	Even with normal pulmonary function values, I believe this person needs a consultation with a pulmonologist with expertise in ILD	91%	44
An asymptomatic patient with either honeycombing or traction bronchiectasis undergoes pulmonary function testing. What reduction from baseline in diffusion capacity do you consider clinically significant warranting a referral to a pulmonologist with expertise in ILD?	Even with normal pulmonary function values, I believe this person needs a consultation with a pulmonologist with expertise in ILD	82%	44
An asymptomatic patient with nondependent subpleural reticulation occupying ≥ 5% of the lung scan (without honeycombing or traction bronchiectasis) has pulmonary function testing. What reduction from baseline in FVC do you consider clinically significant warranting a referral to a pulmonologist with expertise in ILD?	Even with normal pulmonary function values, I believe this person needs a consultation with a pulmonologist with expertise in ILD	77%	44
Screening for early stages of ILD			
Would you recommend screening in an asymptomatic patient aged > 50 years with a history of scleroderma and without crackles on lung auscultation? Screening can include pulmonary function testing and/or CT imaging.	Yes	89%	44
Survey 2			
Diagnosis and reporting of incidentally detected ILA			
In your practice, do you consider each of the following findings to generally indicate the presence of a potentially progressive ILD?			
Honeycombing (irrespective of extent or distribution)	Yes	95%	42
Honeycombing with lower-lobe predominance	Yes	98%	42
Traction bronchiectasis/bronchiolectasis (irrespective of extent or distribution)	Yes	88%	42
Traction bronchiectasis/bronchiolectasis with subpleural reticulation and with a lower-lobe predominance	Yes	93%	42
In your practice, do you consider each of the following findings to generally indicate the presence of potentially progressive fibrosing ILD?			
Honeycombing (irrespective of extent or distribution)	Yes	93%	42
Honeycombing with lower-lobe predominance	Yes	95%	42
Traction bronchiectasis/bronchiolectasis (irrespective of extent or distribution)	Yes	83%	42
Traction bronchiectasis/bronchiolectasis with subpleural reticulation and with a lower-lobe predominance	Yes	93%	42
Centrilobular ground-glass nodules or patchy ground-glass opacity (without honeycombing, traction bronchiectasis or subpleural reticulation)	No	83%	42
Regarding each of the findings below, should the radiology report include the following components?			
S-modifier?			
Honeycombing (irrespective of extent or distribution)	Yes	86%	42
Honeycombing with lower-lobe predominance	Yes	95%	42
Traction bronchiectasis/bronchiolectasis (irrespective of extent or distribution)	Yes	88%	42
Traction bronchiectasis/bronchiolectasis with subpleural reticulation and with a lower-lobe predominance	Yes	90%	42
Recommendation in the report conclusion to refer to pulmonologist?			
Honeycombing (irrespective of extent or distribution)	Yes	79%	42
Honeycombing with lower-lobe predominance	Yes	90%	42
Traction bronchiectasis/bronchiolectasis (irrespective of extent or distribution)	Yes	79%	42
Traction bronchiectasis/bronchiolectasis with subpleural reticulation and with a lower-lobe predominance	Yes	86%	42
Screening for early stages of ILD			
Would you recommend screening in an asymptomatic patient with a history of scleroderma and without crackles on lung auscultation (and does not have diagnosed familial fibrosis)? Screening can include pulmonary function testing and/or CT imaging.	Yes	83%	42

Questions and answers are only shown where consensus (75% agreement or disagreement) was achieved. Refer to e-Tables 1 and 2 for all questions included in the two surveys. S-modifier indicates clinically significant or potentially significant noncancer findings. HRCT = high-resolution CT; ILA = interstitial lung abnormalities; ILD = interstitial lung disease; Lung-RADS = Lung Imaging Reporting and Data System.

#### Perceptions of Significance of ILA

In the initial survey, the expert panel achieved consensus on the following three statements: “in a person without a clinical diagnosis of ILD, ILA are generally defined by the presence of CT imaging features that suggest an ILD”; “research participants with ILA without a diagnosis of ILD have demonstrated similar but often less severe physiologic decrements than those with clinical ILD”; and “research participants with ILA have an increased rate of mortality.”

#### Diagnosis and Reporting of ILA

In the responses to Survey One and as confirmed in Survey Two, there was consensus that honeycombing always indicates the presence of an underlying ILD. In addition, the panel achieved consensus that centrilobular ground-glass nodules or patchy ground-glass opacity, without other findings, do not always indicate the presence of an underlying ILD (Table 2). There was also consensus that on identifying honeycombing on lung cancer screening scans, radiologists should include a Lung CT Screening Reporting and Data System (Lung-RADS) “S-modifier.” Lung-RADS is a quality assurance tool developed by the American College of Radiology to standardize reporting and management recommendations for lung cancer screening. The S-modifier indicates clinically significant or potentially significant noncancer findings.^26^ Relating to this question, the expert panel agreed that the conclusion of radiology reports in which honeycombing is described should recommend consideration for referral to a pulmonologist with experience in ILDs.

**Figure 2:**
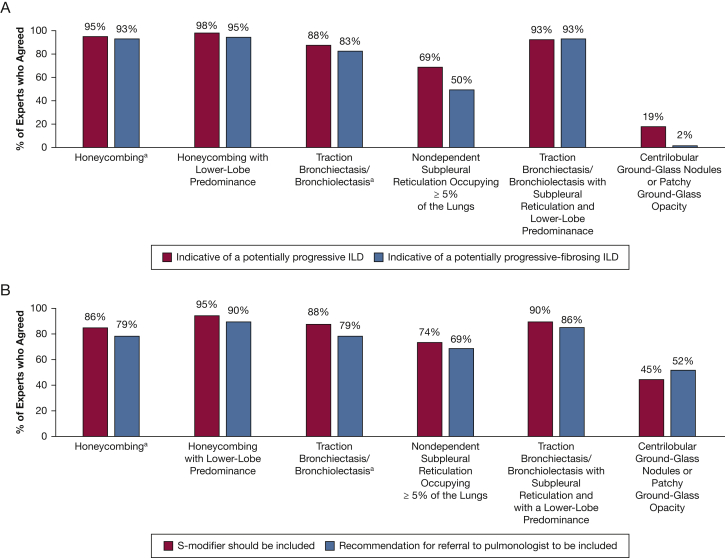
Opinions of experts (n = 42; Survey Two) regarding interstitial lung abnormalities observed on chest CT scans during lung cancer screening: (A) abnormalities that may indicate the presence of ILD; and (B) abnormalities that warrant inclusion of an S-modifier and referral to a pulmonologist. The S-modifier indicates clinically significant or potentially significant noncancer findings. ^a^Irrespective of extent or distribution. ILD = interstitial lung disease.

In Survey Two, there was consensus that honeycombing and traction bronchiectasis or bronchiolectasis indicated potentially progressive ILD. Honeycombing, irrespective of extent or distribution, was believed to indicate the presence of a fibrosing or potentially progressive fibrosing ILD. Likewise, the expert panel thought that traction bronchiectasis and bronchiolectasis were associated with potentially progressive-fibrosing ILD. Agreement on this topic increased from 83% to 93% when this finding was lower-lobe predominant (Table 2). Findings that did not achieve consensus with respect to being indicative of ILD included nondependent subpleural reticulation occupying > 5% of the CT scan (69% agreed) and centrilobular ground-glass nodules (19% agreed) (Fig 2, Table 2).

When interpreting chest CT scans from lung cancer screening in smokers, the expert panel achieved consensus on recommending the use of a Lung-RADS S-modifier when the following findings are identified: honeycombing irrespective of extent or distribution (consensus increased [numerically] from 86% to 95% agreement when the honeycombing was lower-lobe predominant); traction bronchiectasis or bronchiolectasis, irrespective of distribution (consensus increased from 88% to 90% agreement when this finding was lower-lobe predominant) (Table 2). The expert panel also achieved consensus regarding inclusion of a recommendation for referral to a pulmonologist with expertise in ILD in the radiology report, when honeycombing (irrespective of extent or distribution) and traction bronchiectasis or bronchiolectasis are identified (Table 2).

#### Diagnostic Testing in Asymptomatic Patients

**Figure 3:**
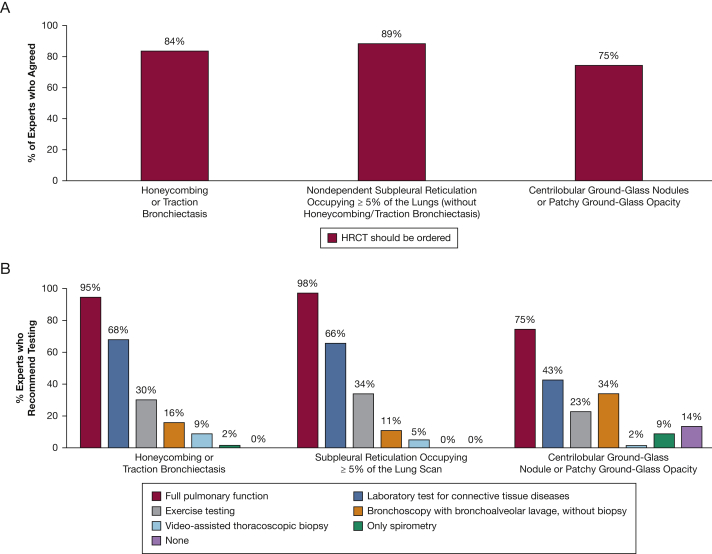
Expert opinions (n = 44; Survey One) regarding practices when asymptomatic patients are referred regarding (A) whether HCRT imaging should be ordered in patients who previously had the indicated CT scan-detected interstitial lung abnormalities; and (B) the types of pulmonary function tests that should be recommended in patients who previously had the same interstitial lung abnormalities detected following HRCT imaging. HRCT = high-resolution CT.

In the initial survey, the expert panel recommended ordering a high-resolution chest CT scan (HRCT), if not already performed, when any of the following findings are present in the screening CT scan: nondependent subpleural reticulation on ≥ 5% of the CT scan; traction bronchiectasis or honeycombing; or centrilobular ground-glass nodules or patchy ground-glass opacity (Fig 3A, Table 2). The expert panel also agreed that full pulmonary function tests (PFTs), including spirometry, lung volumes, and measurement of diffusion capacity, should be performed in people identified with these imaging findings (Fig 3B, Table 2).

Regarding interpretation of PFTs, the panel agreed that even if diffusion capacity measurements were in the normal range, if honeycombing or traction bronchiectasis was present, the patient should be referred to a pulmonologist with expertise in ILD (Table 2).

For patients with confirmed ILA but no PFT abnormalities, > 90% of expert panel members recommended follow-up, specialist referral, or periodic repeat testing. Although consensus was not agreed on any one option, most members of the expert panel agreed that asymptomatic patients with nondependent subpleural reticulation, honeycombing/traction bronchiectasis, or centrilobular ground-glass nodules/patchy ground-glass opacity should be followed up within 6 to 12 months. For all three of these ILA, consensus was not met regarding the type of follow-up testing. Full PFTs were recommended by the majority of clinicians, with split opinions on whether these should be accompanied by HRCT scanning. A minority of respondents recommended HRCT scanning without PFTs.

#### ILA Screening in High-Risk Populations

**Figure 4:**
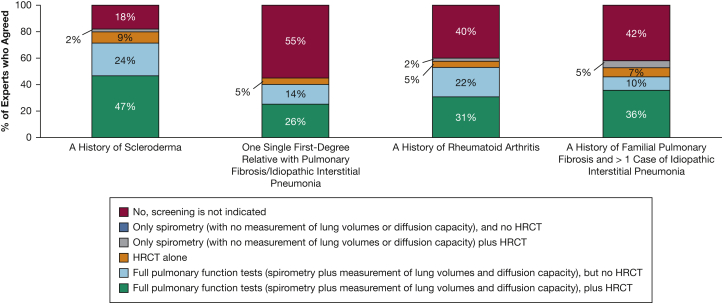
Opinions of experts (n = 42; Survey Two) regarding need for, and type of, screening in asymptomatic patients without crackles on lung auscultation who have the indicated risk factors. HRCT = high-resolution CT.

In Survey One, consensus was achieved (89% agreement) regarding the need for general screening for ILD in patients aged > 50 years in the setting of systemic sclerosis. Substantial agreement (73%) was achieved regarding the need for screening in patients without crackles on lung examination who are undiagnosed but have > 1 relative with an idiopathic interstitial pneumonia. In patients with rheumatoid arthritis but without crackles on lung auscultation, consensus on screening was not achieved (64% agreed). Similar degrees of consensus on screening were achieved in Survey Two; 83% recommended screening for early stages of ILD in patients with systemic sclerosis (Table 2). Of those who recommended screening, most recommended use of full PFTs; however, consensus was not reached on the requirement for accompanying HRCT imaging (Fig 4).

## Discussion

This article presents the results of systematic eliciting of the opinions and recommendations of expert pulmonologists and thoracic radiologists, regarding the reporting, referral and clinical evaluation of patients with ILA, and screening for ILD in at-risk populations. The study describes several areas of consensus. There was consensus that ILA which include honeycombing or traction bronchiectasis regardless of the extent are more likely to represent clinically significant ILD and warrant a Lung-RADS S-modifier,^27^ as well as referral to a pulmonologist with expertise in ILD for clinical assessment. There was also consensus that other forms of ILA should be actively followed up within 1 year. In addition, there was consensus that patients with systemic sclerosis, including those without significant respiratory symptoms or notable physical examination findings, warrant screening for the presence of ILD. These results provide some guidance for chest radiologists seeking to establish standards on reporting ILA (eg, for programs adopting lung cancer screening,^28^ or for assessments of scans performed for other reasons) and for programs considering the appropriateness of screening protocols in high-risk populations.

A major goal of ILA research is to help identify early stages of an undiagnosed form of progressive ILD that may ultimately result in adverse clinical outcomes. Numerous studies in diverse populations have shown that histopathologic findings in research participants with ILA have some overlapping features with those in patients with idiopathic pulmonary fibrosis.^16^ Individuals with ILA can also experience restrictive physiological impairments at rest and on exertion,^11^^,^^15^^,^^18^ radiologic progression,^10^^,^^20^ and accelerated lung function decline.^10^ They may also have an increased risk of death^7^^,^^10^^,^^17^^,^^20^^,^^29^^,^^30^ that is more pronounced among those with fibrotic imaging findings.^20^ Prior to this study, there have been limited data^22^ on the opinions of pulmonologists and thoracic radiologists, who have to report on, and manage, patients with these imaging findings.

The anonymized and iterative survey process described in this article preceded the recent publication of the Fleischner Society position paper on ILA,^22^ which elicited broadly similar conclusions. One important distinction is that our study emphasizes the importance of systematically reporting the presence of ILA with honeycombing or traction bronchiectasis as a clinically significant finding on lung cancer screening scans, so that associated radiology reports should recommend consideration of referral to a pulmonologist. These findings have important implications for thoracic radiologists and their evaluation of lung cancer-screening CT scans. Although the American College of Radiology/Society of Thoracic Radiology guidelines recommend the use of an S-modifier for “clinically significant or potentially clinically significant findings other than lung cancer”,^31^ this term is not defined. Previous studies have reported inconsistent inclusion of Lung-RADS S-modifiers by radiologists for various incidentally detected imaging findings, including coronary arterial calcification^32^ and ILA.^21^ The expert panel in this study achieved consensus that chest findings of honeycombing and traction bronchiectasis likely define an underlying ILD, which warrants an S-modifier and a referral to a pulmonologist for further evaluation. Although a majority believed that these recommendations should also apply to the presence of nondependent subpleural reticulation without honeycombing or traction bronchiectasis, consensus was not reached. Research participants with ILA, defined according to subpleural reticulation, have an increased rate of mortality, compared with those without subpleural reticulation^20^; however, the lack of consensus for defining ILA by subpleural reticulation alone suggests the need for future work evaluating the natural history of subpleural reticulations and their clinical implications.

Our data also provide valuable recommendations for pulmonologists (and rheumatologists) regarding their approach to the follow-up of patients with incidentally observed ILA. The data may also better inform pulmonologists’ perception and understanding regarding the value of chest CT screening in the at-risk populations they manage. Although there were differences of opinions regarding the recommended follow-up practices for patients with most forms of incidentally identified ILA, lung biopsy was rarely recommended as part of the initial evaluation.

There was consensus that patients with systemic sclerosis need further screening to detect possible signs and symptoms of early-stage ILD (as opposed to ILA, which are radiologic observations); 85% of respondents recommended PFTs that included measures of gas exchange. In contrast, however, opinions were split on whether radiologic imaging with chest CT imaging should also be recommended alongside PFTs. Given the differences of opinion on screening in other at-risk populations, future research studies on screening in these populations may be warranted.

The strengths of our study include the consolidation of the opinions and advice of experts with substantial expertise; the integration of opinions from both radiologists and pulmonologists; the iterative approach to refine and build upon initial findings; and the international nature of the expert panel members. However, there are important limitations to these findings and their interpretation. The hope that early identification of those with, or at-risk for, progressive pulmonary fibrosis will lead to earlier interventions that could improve outcomes is not yet proven. In addition, although consensus was reached that HRCT scans were recommended as a follow-up to screening evaluations, it is important for clinicians to review individual patient histories to avoid ordering unnecessary duplicate HRCT imaging. Moreover, although promising literature indicates that antifibrotic therapy can reduce the rate of lung-function decline in patients with idiopathic pulmonary fibrosis^33^^,^^34^ and other forms of progressive fibrosing ILDs,^35^, ^36^, ^37^ including those with preserved lung function,^38^, ^39^, ^40^, ^41^ patients with early-stage disease might also benefit from nonmedical interventions (eg, smoking cessation and occupational or environmental risk reduction).^42^^,^^43^

Some of our recommendations may increase the burden to physicians during patient visits and the emotional distress to patients. In addition, the opinions of the experts who agreed to participate in these surveys may not be reflective of the broader community. In community practice settings in which chest CT screening scans are commonly interpreted by general radiologists who may have less experience in ILA interpretation, additional training may be necessary prior to considering adoption of these consensus recommendations. In our approach, we chose to pose most questions with categorical answer options to permit clear identification of opinions, but greater use of continuous response scales (eg, Likert), particularly in the first survey, may have provided greater subtlety of response. Also, respondents had limited exposure to questions during the initiative, with a number of unique questions only answered once by respondents. Additional rounds of surveying with more repeating or rephrased questions may have helped to further build consensus. Furthermore, sensitivity analyses were not part of our a priori definition of consensus; these may have added further confidence to our findings. Finally, consensus recommendations on patient evaluations and referrals were largely based on current knowledge and thus may change with future publications.

## Interpretation

There was consensus among members of an expert panel that chest CT evidence of honeycombing or traction bronchiectasis/bronchiolectasis likely defines a potentially progressive ILD and warrants reporting as a clinically significant finding on lung cancer screening CT scans, as well as a referral to a pulmonologist with ILD expertise. In addition, there was consensus that patients with systemic sclerosis should undergo screening for the presence of ILD. Our findings may provide additional guidance for thoracic radiologists, as well as those involved in clinical programs who are considering screening for early ILD detection; however, these findings should be confirmed by further rigorous clinical investigations.

**Take-home Points**

**Study Question:** What do experts recommend as best practice to guide and inform clinicians in the recognition, referral, and follow-up of patients with, or at risk of developing, early ILDs?

**Results:** Two rounds of iterative surveys found consensus among international experts that CT evidence of honeycombing or traction bronchiectasis probably defines the presence of progressive ILD and warrants reporting as a potentially significant finding and referral to a pulmonologist with expertise in ILD.

**Interpretation:** Guidance recommending early identification and follow-up of ILA was established and is expected to improve outcomes for patients with such findings.

## Supplementary Data

e-Online Data
