# Supplementary material for: Detection and Early Referral of Patients With Interstitial Lung Abnormalities: An Expert Survey Initiative
Source: Chest. 2021 Jun 29;161(2):470–82. doi: 10.1016/j.chest.2021.06.035 (PMC10624930; doi:10.1016/j.chest.2021.06.035)
Supplement: e-Online Data [file mmc1.pdf]

CHEST Online Supplement

Detection and Early Referral of Patients With Interstitial Lung Abnormalities

An Expert Survey Initiative

Gary M. Hunninghake, MD, MPH; Jonathan G. Goldin, MD, PhD; Michael A. Kadoch, MD; Jonathan A. Kropski, MD; Ivan O. Rosas, MD; Athol U. Wells, MD, PhD; Ruchi Yadav, MD; Howard M. Lazarus, MD; Fereidoun G. Abtin, MD; Tamera J. Corte, MD, PhD; Joao A. de Andrade, MD; Kerri A. Johannson, MD, MPH; Martin R. Kolb, MD, PhD; David A. Lynch, MB, BCh; Justin M. Oldham, MD; Paolo Spagnolo, MD, PhD; Mary E. Strek, MD; Sara Tomassetti, MD; George R. Washko, MD; and Eric S. White, MD; on behalf of the ILA Study Group

CHEST 2022; 161(2):470-482

Online supplements are not copyedited prior to posting and the author(s) take full responsibility for the accuracy of all data.

© 2022 AMERICAN COLLEGE OF CHEST PHYSICIANS. Reproduction of this article is prohibited without written permission from the American College of Chest Physicians. See online for more details. **DOI:** 10.1016/j.chest.2021.06.035

e-Table 1 Survey One Clinical Questions and Responses

Question	Number of respondents per question[a]	Options available	Agreement per option, %
Perceptions of ILA			
In a person without a clinical diagnosis of ILD, ILA are generally defined by the presence of chest-CT imaging features that suggest an underlying ILD: agree or disagree?	44	Agree	84%
		Disagree	14%
		Unsure	2%
Undiagnosed research participants with ILA have demonstrated similar, but often less severe, physiologic decrements than those noted on patients with clinically apparent ILD: agree or disagree?	44	Agree	80%
		Disagree	11%
		Unsure	9%
Undiagnosed research participants with ILA have demonstrated an increased rate of mortality: agree or disagree?	44	Agree	77%
		Disagree	11%
		Unsure	11%
Diagnosis and reporting of incidentally detected ILA			
In your practice, do you consider honeycombing (irrespective of its extent or distribution) to always indicate the presence of ILD?	44	Yes	75%
		No	20%
		Unsure	5%
[If Yes or Unsure] Should radiologists include a Lung-RADS™ "S modifier" regarding this finding in their report of lung cancer screening CT scans?	35	Yes	86%
		No	6%
		Unsure	9%
[If Yes or Unsure] Should the conclusion of the radiology report also recommend	33	Yes	76%

Online supplements are not copyedited prior to posting and the author(s) take full responsibility for the accuracy of all data.

consideration of a referral to a pulmonologist?		No	3%
		Unsure	21%
In your practice, do you consider honeycombing (irrespective of its extent or distribution) to always indicate the presence of a fibrosing ILD?	44	Yes	75%
		No	18%
		Unsure	7%
[If Yes or Unsure] Should radiologists include a Lung-RADS™ "S modifier" regarding this finding in their report of lung cancer screening CT scans?	36	Yes	86%
		No	0%
		Unsure	14%
[If Yes or Unsure] Should the conclusion of the radiology report also recommend consideration of a referral to a pulmonologist?	36	Yes	78%
		No	8%
		Unsure	14%
In your practice, do you consider traction bronchiectasis/bronchiolectasis (irrespective of its extent or distribution) to always indicate the presence of ILD?	44	Yes	50%
		No	45%
		Unsure	5%
[If Yes or Unsure] Should radiologists include a Lung-RADS™ "S modifier" regarding this finding in their report of lung cancer screening CT scans?	24	Yes	83%
		No	0%
		Unsure	17%
[If Yes or Unsure] Should the conclusion of the radiology report also recommend consideration of a referral to a pulmonologist?	24	Yes	83%
		No	4%

Online supplements are not copyedited prior to posting and the author(s) take full responsibility for the accuracy of all data.

		Unsure	13%
In your practice, do you consider traction bronchiectasis/bronchiolectasis (irrespective of its extent or distribution) to always indicate the presence of fibrosing ILD?	44	Yes	52%
		No	43%
		Unsure	5%
[If Yes or Unsure] Should radiologists include a Lung-RADS™ "S modifier" regarding this finding in their report of lung cancer screening CT scans?	25	Yes	80%
		No	4%
		Unsure	16%
[If Yes or Unsure] Should the conclusion of the radiology report also recommend consideration of a referral to a pulmonologist?	24	Yes	79%
		No	4%
		Unsure	17%
In your practice, do you consider non-dependent subpleural reticulation occupying ≥ 5% of the lung scan (without honeycombing or traction bronchiectasis) to always indicate the presence of ILD?	44	Yes	45%
		No	41%
		Unsure	14%
[If Yes or Unsure] Should radiologists include a Lung-RADS™ "S modifier" regarding this finding in their report of lung cancer screening CT scans?	26	Yes	85%
		No	4%
		Unsure	12%
[If Yes or Unsure] Should the conclusion of the radiology report also recommend consideration of a referral to a pulmonologist?	25	Yes	76%
		No	4%
		Unsure	20%

Online supplements are not copyedited prior to posting and the author(s) take full responsibility for the accuracy of all data.

In your practice, do you consider non-dependent subpleural reticulation occupying ≥ 5% of the lung scan (without honeycombing or traction bronchiectasis) to always indicate the presence of fibrosing ILD?	44	Yes	27%
		No	64%
		Unsure	9%
[If Yes or Unsure] Should radiologists include a Lung-RADS™ "S modifier" regarding this finding in their report of lung cancer screening CT scans?	16	Yes	88%
		No	6%
		Unsure	6%
[If Yes or Unsure] Should the conclusion of the radiology report also recommend consideration of a referral to a pulmonologist?	15	Yes	87%
		No	0%
		Unsure	13%
In your practice, do you consider centrilobular ground-glass nodules or patchy ground-glass opacity (without honeycombing, traction bronchiectasis or significant subpleural reticulation) to always indicate the presence of ILD?	44	Yes	5%
		No	93%
		Unsure	2%
[If Yes or Unsure] Should radiologists include a Lung-RADS™ "S modifier" regarding this finding in their report of lung cancer screening CT scans?	3	Yes	100%
		No	0%
		Unsure	0%
[If Yes or Unsure] Should the conclusion of the radiology report also recommend consideration of a referral to a pulmonologist?	3	Yes	67%
		No	0%
		Unsure	33%
In your practice, do you consider centrilobular ground-glass nodules or patchy ground-glass	44	Yes	2%

Online supplements are not copyedited prior to posting and the author(s) take full responsibility for the accuracy of all data.

opacity (without honeycombing, traction bronchiectasis or significant subpleural reticulation) to always indicate the presence of fibrosing ILD?		No	95%
		Unsure	2%
[If Yes or Unsure] Should radiologists include a Lung-RADS™ "S modifier" regarding this finding in their report of lung cancer screening CT scans?	2	Yes	50%
		No	0%
		Unsure	50%
[If Yes or Unsure] Should the conclusion of the radiology report also recommend consideration of a referral to a pulmonologist?	2	Yes	100%
		No	0%
		Unsure	0%
Testing of referred asymptomatic patients			
Please consider the clinical approach to the diagnosis of ILD or fibrosing ILD in the future. How important do you think that quantitative CT imaging (using computerized methods) will be?	44	1 (Not important at all)	0%
		2	9%
		3	18%
		4	32%
		5 (Extremely important)	41%
		Unsure	0%
Please consider the clinical approach to defining the extent of ILD and the longitudinal follow-up of disease in the future. How important do you think that quantitative CT imaging (using computerized methods) will be?	44	1 (Not important at all)	0%
		2	0%
		3	14%
		4	43%

Online supplements are not copyedited prior to posting and the author(s) take full responsibility for the accuracy of all data.

		5 (Extremely important)	43%
		Unsure	0%
An asymptomatic patient is referred to clinic based on the presence of either honeycombing or traction bronchiectasis on a CT. If not done already, should HRCT be ordered?	44	Yes	84%
		No	11%
		Maybe	5%
An asymptomatic patient is referred to clinic based on the presence of either honeycombing or traction bronchiectasis on a CT. Which of the following tests, if any, would you order in your practice?	44	No tests are indicated	0%
		Only spirometry (without lung volumes or measurement of diffusion capacity)	2%
		Full pulmonary function tests (spirometry plus lung volumes and measurement of diffusion capacity)	95%
		Exercise testing	30%
		Laboratory testing for connective tissue diseases	68%
		Bronchoscopy with bronchoalveolar lavage, without a lung biopsy	16%
		Video-assisted thoracoscopic lung biopsy	9%
An asymptomatic patient is referred to clinic based on the presence of subpleural reticulation occupying ≥ 5% of the lung scan (without honeycombing or traction bronchiectasis). If not done already, should HRCT be ordered?	44	Yes	89%
		No	9%
		Unsure	2%

Online supplements are not copyedited prior to posting and the author(s) take full responsibility for the accuracy of all data.

An asymptomatic patient is referred to clinic based on the presence of subpleural reticulation occupying ≥ 5% of the lung scan (without honeycombing or traction bronchiectasis) on HRCT. Which of the following tests, if any, would you order in your practice?	44	No tests are indicated	0%
		Only spirometry (without lung volumes or measurement of diffusion capacity)	0%
		Full pulmonary function tests (spirometry plus lung volumes and measurement of diffusion capacity)	98%
		Exercise testing	34%
		Laboratory testing for connective tissue diseases	66%
		Bronchoscopy with bronchoalveolar lavage, without a lung biopsy	11%
		Video-assisted thoracoscopic lung biopsy	5%
An asymptomatic patient is referred to clinic based on the presence of centrilobular ground-glass nodules or patchy ground-glass opacity (without honeycombing, traction bronchiectasis or significant subpleural reticulation). If not done already, should a HRCT be ordered?	44	Yes	75%
		No	23%
		Unsure	2%
An asymptomatic patient is referred to clinic based on the presence of centrilobular ground-glass nodules or patchy ground-glass opacity (without honeycombing, traction bronchiectasis or significant subpleural reticulation) on HRCT. Which of the following tests, if any, would you order in your practice?	44	No tests are indicated	14%
		Only spirometry (without lung volumes or measurement of diffusion capacity)	9%
		Full pulmonary function tests (spirometry plus lung volumes	75%

Online supplements are not copyedited prior to posting and the author(s) take full responsibility for the accuracy of all data.

		and measurement of diffusion capacity)	
		Exercise testing	23%
		Laboratory testing for connective tissue diseases	43%
		Bronchoscopy with bronchoalveolar lavage, without a lung biopsy	34%
		Video-assisted thoracoscopic lung biopsy	2%
An asymptomatic patient with either traction bronchiectasis or honeycombing undergoes full pulmonary function testing (no prior pulmonary function testing is available) and does not demonstrate significant abnormalities based on predefined significance thresholds.<br>What would you recommend next?	44	No further evaluation is needed	2%
		The patient should be recommended for a lung biopsy	2%
		The patient should not be recommended initially for a lung biopsy, but they should be evaluated by a pulmonologist with expertise in ILD	61%
		The patient should be followed up with periodic repeat testing before any decision on a lung biopsy or pulmonary referral is made	34%
An asymptomatic patient with non-dependent subpleural reticulation occupying ≥ 5% of the lung scan (without honeycombing or traction bronchiectasis) undergoes full pulmonary function testing (no prior pulmonary function testing is available) and does not demonstrate significant abnormalities based on predefined	44	No further evaluation is needed	2%
		The patient should be recommended for a lung biopsy	5%
		The patient should not be recommended initially for a lung biopsy, but they should be	52%

Online supplements are not copyedited prior to posting and the author(s) take full responsibility for the accuracy of all data.

significance thresholds. What would you recommend next?		evaluated by a pulmonologist with expertise in ILD	
		The patient should be followed up with periodic repeat testing before any decision on a lung biopsy or pulmonary referral is made	41%
An asymptomatic patient with centrilobular ground-glass nodules or patchy ground-glass opacity (without honeycombing, traction bronchiectasis or significant subpleural reticulation) undergoes full pulmonary function testing (no prior pulmonary function testing is available), and does not demonstrate significant abnormalities based on predefined significance thresholds. What would you recommend next?	44	No further evaluation is needed	9%
		The patient should be recommended for a lung biopsy	2%
		The patient should not be recommended initially for a lung biopsy, but they should be evaluated by a pulmonologist with expertise in ILD	48%
		The patient should be followed up with periodic repeat testing before any decision on a lung biopsy or pulmonary referral is made	41%
Interpreting pulmonology tests			
An asymptomatic patient with either honeycombing or traction bronchiectasis undergoes pulmonary function testing. What reduction from baseline in forced vital capacity do you consider clinically significant warranting a referral to a pulmonologist with expertise in ILD?	44	Without symptoms, I would not take action on this finding alone	0%
		Even with normal pulmonary function values, I believe this person needs a consultation with a pulmonologist with expertise in ILD	91%
		Even with normal pulmonary function values, I believe this person needs a consultation with a pulmonologist with expertise in ILD	7%

Online supplements are not copyedited prior to posting and the author(s) take full responsibility for the accuracy of all data.

		< 70% of predicted	2%
		< 60% of predicted	0%
An asymptomatic patient with either honeycombing or traction bronchiectasis undergoes pulmonary function testing. What reduction from baseline in diffusion capacity do you consider clinically significant warranting a referral to a pulmonologist with expertise in ILD?	44	Without symptoms, I would not take action on this finding alone	0%
		Even with normal pulmonary function values, I believe this person needs a consultation with a pulmonologist with expertise in ILD	82%
		Even with normal pulmonary function values, I believe this person needs a consultation with a pulmonologist with expertise in ILD	14%
		< 70% of predicted	2%
		< 60% of predicted	2%
An asymptomatic patient with non-dependent subpleural reticulation occupying ≥ 5% of the lung scan (without honeycombing or traction bronchiectasis) has pulmonary function testing. What reduction from baseline in forced vital capacity do you consider clinically significant warranting a referral to a pulmonologist with expertise in ILD?	44	Without symptoms, I would not take action on this finding alone	0%
		Even with normal pulmonary function values, I believe this person needs a consultation with a pulmonologist with expertise in ILD	77%
		< 80% of predicted (or <LLN)	18%
		< 70% of predicted	5%

Online supplements are not copyedited prior to posting and the author(s) take full responsibility for the accuracy of all data.

		< 60% of predicted	0%
An asymptomatic patient with non-dependent subpleural reticulation occupying at least 5% of the lung scan (without honeycombing or traction bronchiectasis) has pulmonary function testing. What reduction from baseline in diffusion capacity do you consider clinically significant warranting a referral to a pulmonologist with expertise in ILD?	44	Without symptoms, I would not take action on this finding alone	2%
		Even with normal pulmonary function values, I believe this person needs a consultation with a pulmonologist with expertise in ILD	68%
		Even with normal pulmonary function values, I believe this person needs a consultation with a pulmonologist with expertise in ILD	23%
		< 70% of predicted	2%
		< 60% of predicted	5%
An asymptomatic patient with centrilobular ground-glass nodules or patchy ground-glass opacity (without honeycombing, traction bronchiectasis or significant subpleural reticulation) has pulmonary function testing. What reduction from baseline in forced vital capacity do you consider clinically significant warranting a referral to a pulmonologist with expertise in ILD?	44	Without symptoms, I would not take action on this finding alone	5%
		Even with normal pulmonary function values, I believe this person needs a consultation with a pulmonologist with expertise in ILD	55%
		Even with normal pulmonary function values, I believe this person needs a consultation with a pulmonologist with expertise in ILD	32%
		< 70% of predicted	7%
		< 60% of predicted	2%

Online supplements are not copyedited prior to posting and the author(s) take full responsibility for the accuracy of all data.

An asymptomatic patient with centrilobular ground-glass nodules or patchy ground-glass opacity (without honeycombing, traction bronchiectasis or significant subpleural reticulation) has pulmonary function testing. What reduction from baseline in diffusion capacity do you consider clinically significant warranting a referral to a pulmonologist with expertise in ILD?	44	Without symptoms, I would not take action on this finding alone	7%
		Even with normal pulmonary function values, I believe this person needs a consultation with a pulmonologist with expertise in ILD	50%
		Even with normal pulmonary function values, I believe this person needs a consultation with a pulmonologist with expertise in ILD	32%
		< 70% of predicted	5%
		< 60% of predicted	7%
Follow-up requirements			
An asymptomatic patient with either honeycombing or traction bronchiectasis has no measurable decrements on pulmonary function testing (no prior pulmonary function testing is available). When would you recommend following this patient up in your practice?	44	No further follow up is needed	2%
		In 6 months	68%
		In 1 year	27%
		In ≥ 2 years	2%
What follow-up testing would you recommend?	43	Only spirometry (with no measurement of lung volumes or diffusion capacity), and no HRCT	2%
		Full pulmonary function tests (spirometry plus measurement of lung volumes and diffusion capacity), but no HRCT	47%
		HRCT alone	7%

Online supplements are not copyedited prior to posting and the author(s) take full responsibility for the accuracy of all data.

		Only spirometry (with no measurement of lung volumes or diffusion capacity) plus HRCT	7%
		Full pulmonary function tests (spirometry plus measurement of lung volumes and diffusion capacity), plus HRCT	37%
An asymptomatic patient with non-dependent subpleural reticulation occupying ≥ 5% of the lung scan (without honeycombing or traction bronchiectasis) has no measurable decrements on pulmonary function testing (no prior pulmonary function testing is available). When would you recommend following this patient up in your practice?	44	No further follow up is needed	2%
		In 6 months	59%
		In 1 year	34%
		In ≥ 2 years	5%
What follow-up testing would you recommend?	43	Only spirometry (with no measurement of lung volumes or diffusion capacity), and no HRCT	2%
		Full pulmonary function tests (spirometry plus measurement of lung volumes and diffusion capacity), but no HRCT	51%
		HRCT alone	12%
		Only spirometry (with no measurement of lung volumes or diffusion capacity) plus HRCT	7%
		Full pulmonary function tests (spirometry plus measurement of lung volumes and diffusion capacity), plus HRCT	28%

Online supplements are not copyedited prior to posting and the author(s) take full responsibility for the accuracy of all data.

An asymptomatic patient with centrilobular ground-glass nodules or patchy ground-glass opacity (without honeycombing, traction bronchiectasis or significant subpleural reticulation) has no measurable decrements on pulmonary function testing (no prior pulmonary function testing is available). When would you recommend following this patient up in your practice?	44	No further follow up is needed	11%
		In 6 months	61%
		In 1 year	25%
		In ≥ 2 years	2%
What follow-up testing would you recommend?	39	Only spirometry (with no measurement of lung volumes or diffusion capacity), and no HRCT	0%
		Full pulmonary function tests (spirometry plus measurement of lung volumes and diffusion capacity), but no HRCT	33%
		HRCT alone	28%
		Only spirometry (with no measurement of lung volumes or diffusion capacity) plus HRCT	3%
		Full pulmonary function tests (spirometry plus measurement of lung volumes and diffusion capacity), plus HRCT	36%
Screening for early stages of ILD			
Would you recommend screening in an asymptomatic patient aged > 50 years with a history of rheumatoid arthritis and without crackles on lung auscultation? Screening can include pulmonary function testing and/or CT imaging.	44	No, screening is not indicated	16%
		Yes	64%
		Unsure	20%

Online supplements are not copyedited prior to posting and the author(s) take full responsibility for the accuracy of all data.

[If Yes or Unsure] Which test(s) would you recommend?		Only spirometry (with no measurement of lung volumes or diffusion capacity), and no HRCT	0%
		Full pulmonary function tests (spirometry plus measurement of lung volumes and diffusion capacity), but no HRCT	32%
		HRCT alone	11%
		Only spirometry (with no measurement of lung volumes or diffusion capacity) plus HRCT	0%
		Full pulmonary function tests (spirometry plus measurement of lung volumes and diffusion capacity), plus HRCT	57%
Would you recommend screening in an asymptomatic patient aged > 50 years with a history of scleroderma and without crackles on lung auscultation? Screening can include pulmonary function testing and/or CT imaging.	44	No, screening is not indicated	7%
		Yes	89%
		Unsure	5%
[If Yes or Unsure] Which test(s) would you recommend?	39	Only spirometry (with no measurement of lung volumes or diffusion capacity), and no HRCT	0%
		Full pulmonary function tests (spirometry plus measurement of lung volumes and diffusion capacity), but no HRCT	26%
		HRCT alone	10%

Online supplements are not copyedited prior to posting and the author(s) take full responsibility for the accuracy of all data.

		Only spirometry (with no measurement of lung volumes or diffusion capacity) plus HRCT	3%
		Full pulmonary function tests (spirometry plus measurement of lung volumes and diffusion capacity), plus HRCT	62%
Would you recommend screening in an asymptomatic patient aged > 50 years with a history of familial pulmonary fibrosis and > 1 case of idiopathic interstitial pneumonia in the family and without crackles on lung auscultation? Screening can include pulmonary function testing and/or CT imaging.	44	No, screening is not indicated	11%
		Yes	73%
		Unsure	16%
[If Yes or Unsure] Which test(s) would you recommend?	32	Only spirometry (with no measurement of lung volumes or diffusion capacity), and no HRCT	3%
		Full pulmonary function tests (spirometry plus measurement of lung volumes and diffusion capacity), but no HRCT	9%
		HRCT alone	16%
		Only spirometry (with no measurement of lung volumes or diffusion capacity) plus HRCT	0%
		Full pulmonary function tests (spirometry plus measurement of lung volumes and diffusion capacity), plus HRCT	72%
Would you recommend screening in an asymptomatic patient aged >50 years who	44	No, screening is not indicated	34%

Online supplements are not copyedited prior to posting and the author(s) take full responsibility for the accuracy of all data.

has one single first-degree relative with pulmonary fibrosis/idiopathic interstitial pneumonia in the family and without crackles on lung auscultation? Screening can include pulmonary function testing and/or CT imaging.		Yes	45%
		Unsure	20%
[If Yes or Unsure] Which test(s) would you recommend?	20	Only spirometry (with no measurement of lung volumes or diffusion capacity), and no HRCT	5%
		Full pulmonary function tests (spirometry plus measurement of lung volumes and diffusion capacity), but no HRCT	25%
		HRCT alone	20%
		Only spirometry (with no measurement of lung volumes or diffusion capacity) plus HRCT	0%
		Full pulmonary function tests (spirometry plus measurement of lung volumes and diffusion capacity), plus HRCT	50%

[a]Demographics- and free text-related questions are not shown.
CT = computed tomography; HRCT = high-resolution computed tomography; ILA = interstitial lung abnormalities; ILD = interstitial lung disease; Lung-RADS™ = Lung imaging reporting and data system; S-modifier = potentially significant incidental findings.

Online supplements are not copyedited prior to posting and the author(s) take full responsibility for the accuracy of all data.

e-Table 2 Survey Two Clinical Questions and Responses

Question	Respondents per question[a]	All available responses	Agreement per option, %
In your practice, do you consider each of the following findings to generally indicate the presence of a potentially progressive ILD?			
Honeycombing (irrespective of extent or distribution)	42	Yes	95%
		No	2%
		Unsure	2%
Honeycombing with lower-lobe predominance	42	Yes	98%
		No	0%
		Unsure	2%
Traction bronchiectasis/bronchiolectasis (irrespective of extent or distribution)	42	Yes	88%
		No	5%
		Unsure	7%
Non-dependent subpleural reticulation occupying ≥ 5% of the lung scan (without honeycombing or traction bronchiectasis)	42	Yes	69%
		No	5%
		Unsure	26%
Traction bronchiectasis/bronchiolectasis with subpleural reticulation and with a lower-lobe predominance	42	Yes	93%
		No	0%
		Unsure	7%
	42	Yes	19%

Online supplements are not copyedited prior to posting and the author(s) take full responsibility for the accuracy of all data.

Centrilobular ground-glass nodules or patchy ground-glass opacity (without honeycombing, traction bronchiectasis or subpleural reticulation)		No	62%
		Unsure	19%
In your practice, do you consider each of the following findings to generally indicate the presence of potentially progressive fibrosing ILD?			
Honeycombing (irrespective of extent or distribution)	42	Yes	93%
		No	2%
		Unsure	5%
Honeycombing with lower-lobe predominance	42	Yes	95%
		No	2%
		Unsure	2%
Traction bronchiectasis/bronchiolectasis (irrespective of extent or distribution)	42	Yes	83%
		No	7%
		Unsure	10%
Non-dependent subpleural reticulation occupying ≥ 5% of the lung scan (without honeycombing or traction bronchiectasis)	42	Yes	50%
		No	17%
		Unsure	33%
Traction bronchiectasis/bronchiolectasis with subpleural reticulation and with a lower lobe predominance	42	Yes	93%
		No	2%
		Unsure	5%

Online supplements are not copyedited prior to posting and the author(s) take full responsibility for the accuracy of all data.

Centrilobular ground-glass nodules or patchy ground-glass opacity (without honeycombing, traction bronchiectasis or subpleural reticulation)	42	Yes	2%
		No	83%
		Unsure	14%
Regarding each of the findings below, should the radiology report include the following components?			
S-modifier?			
Honeycombing (irrespective of extent or distribution)	42	Yes	86%
		No	5%
		Unsure	10%
Honeycombing with lower-lobe predominance	42	Yes	95%
		No	0%
		Unsure	5%
Traction bronchiectasis/bronchiolectasis (irrespective of extent or distribution)	42	Yes	88%
		No	5%
		Unsure	7%
Non-dependent subpleural reticulation occupying ≥ 5% of the lung scan (without honeycombing or traction bronchiectasis)	42	Yes	74%
		No	12%
		Unsure	14%
Traction bronchiectasis/bronchiolectasis with subpleural reticulation and with a lower-lobe predominance	42	Yes	90%
		No	2%

Online supplements are not copyedited prior to posting and the author(s) take full responsibility for the accuracy of all data.

		Unsure	7%
Centrilobular ground-glass nodules or patchy ground-glass opacity (without honeycombing, traction bronchiectasis or subpleural reticulation)	42	Yes	45%
		No	36%
		Unsure	19%
Recommendation in the report conclusion to refer to pulmonologist?			
Centrilobular ground-glass nodules or patchy ground-glass opacity (without honeycombing, traction bronchiectasis or subpleural reticulation)	42	Yes	45%
		No	36%
		Unsure	19%
Honeycombing (irrespective of extent or distribution)	42	Yes	79%
		No	10%
		Unsure	12%
Honeycombing with lower-lobe predominance	42	Yes	90%
		No	2%
		Unsure	7%
Traction bronchiectasis/bronchiolectasis (irrespective of extent or distribution)	42	Yes	79%
		No	10%
		Unsure	12%
Non-dependent subpleural reticulation occupying ≥ 5% of the lung scan (without honeycombing or traction bronchiectasis)	42	Yes	69%
		No	14%

Online supplements are not copyedited prior to posting and the author(s) take full responsibility for the accuracy of all data.

		Unsure	17%
Traction bronchiectasis/bronchiolectasis with subpleural reticulation and with a lower-lobe predominance	42	Yes	86%
		No	5%
		Unsure	10%
Centrilobular ground-glass nodules or patchy ground-glass opacity (without honeycombing, traction bronchiectasis or subpleural reticulation)	42	Yes	52%
		No	26%
		Unsure	21%
An asymptomatic patient with either traction bronchiectasis or honeycombing undergoes full pulmonary function testing (no prior pulmonary function testing is available) and does not demonstrate significant abnormalities based on predefined significance thresholds. What would you recommend next?	42	No further evaluation is needed	2%
		The patient should be recommended for a lung biopsy	0%
		The patient should not be recommended initially for a lung biopsy, but should be evaluated by a pulmonologist with expertise in ILD	71%
		The patient should be recommended for a lung biopsy and should be evaluated by a pulmonologist with expertise in ILD	2%
		The patient should be followed up with periodic repeat testing at least	24%

Online supplements are not copyedited prior to posting and the author(s) take full responsibility for the accuracy of all data.

		annually before any decision on a lung biopsy or pulmonary referral is made	
An asymptomatic patient with non-dependent subpleural reticulation occupying ≥ 5% of the lung scan (without honeycombing or traction bronchiectasis) undergoes full pulmonary function testing (no prior pulmonary function testing is available) and does not demonstrate significant abnormalities based on predefined significance thresholds.<br>What would you recommend next?	42	No further evaluation is needed	2%
		The patient should be recommended for a lung biopsy	0%
		The patient should not be recommended initially for a lung biopsy, but should be evaluated by a pulmonologist with expertise in ILD	60%
		The patient should be recommended for a lung biopsy and should be evaluated by a pulmonologist with expertise in ILD	5%
		The patient should be followed up with periodic repeat testing at least annually before any decision on a lung biopsy or pulmonary referral is made	33%
An asymptomatic patient with centrilobular ground-glass nodules or patchy ground-glass opacity (without honeycombing, traction bronchiectasis or significant subpleural reticulation) undergoes full pulmonary function testing (no prior pulmonary function testing is available), and does not demonstrate significant abnormalities based on	42	No further evaluation is needed	12%
		The patient should be recommended for a lung biopsy	0%

Online supplements are not copyedited prior to posting and the author(s) take full responsibility for the accuracy of all data.

predefined significance thresholds. What would you recommend next?		The patient should not be recommended initially for a lung biopsy, but should be evaluated by a pulmonologist with expertise in ILD	52%
		The patient should be recommended for a lung biopsy and should be evaluated by a pulmonologist with expertise in ILD	2%
		The patient should be followed up with periodic repeat testing at least annually before any decision on a lung biopsy or pulmonary referral is made	33%
Would you recommend screening in an asymptomatic patient with a history of rheumatoid arthritis and without crackles on lung auscultation? Screening can include pulmonary function testing and/or CT imaging.	42	No, screening is not indicated	17%
		Yes	60%
		Unsure	24%
[If Yes or Unsure] Which test(s) would you recommend?		Only spirometry (with no measurement of lung volumes or diffusion capacity), and no HRCT	0%
		Full pulmonary function tests (spirometry plus measurement of lung volumes and diffusion capacity), but no HRCT	36%

Online supplements are not copyedited prior to posting and the author(s) take full responsibility for the accuracy of all data.

		HRCT alone	8%
		Only spirometry (with no measurement of lung volumes or diffusion capacity) plus HRCT	4%
		Full pulmonary function tests (spirometry plus measurement of lung volumes and diffusion capacity), plus HRCT	52%
Would you recommend screening in an asymptomatic patient with a history of scleroderma and without crackles on lung auscultation (and does not have diagnosed familial fibrosis)? Screening can include pulmonary function testing and/or CT imaging.	42	No, screening is not indicated	7%
		Yes	83%
		Unsure	10%
[If Yes or Unsure] Which test(s) would you recommend?	35	Only spirometry (with no measurement of lung volumes or diffusion capacity), and no HRCT	0%
		Full pulmonary function tests (spirometry plus measurement of lung volumes and diffusion capacity), but no HRCT	29%
		HRCT alone	11%
		Only spirometry (with no measurement of lung volumes or diffusion capacity) plus HRCT	3%

Online supplements are not copyedited prior to posting and the author(s) take full responsibility for the accuracy of all data.

		Full pulmonary function tests (spirometry plus measurement of lung volumes and diffusion capacity), plus HRCT	57%
Would you recommend screening in an asymptomatic patient with a history of familial pulmonary fibrosis and > 1 case of idiopathic interstitial pneumonia in the family, and without crackles on lung auscultation? Screening can include pulmonary function testing and/or CT imaging.	42	No, screening is not indicated	21%
		Yes	57%
		Unsure	21%
[If Yes or Unsure] Which test(s) would you recommend?		Only spirometry (with no measurement of lung volumes or diffusion capacity), and no HRCT	0%
		Full pulmonary function tests (spirometry plus measurement of lung volumes and diffusion capacity), but no HRCT	17%
		HRCT alone	13%
		Only spirometry (with no measurement of lung volumes or diffusion capacity) plus HRCT	8%
		Full pulmonary function tests (spirometry plus measurement of lung volumes and diffusion capacity), plus HRCT	63%

Online supplements are not copyedited prior to posting and the author(s) take full responsibility for the accuracy of all data.

Would you consider screening in an asymptomatic patient who has one single first-degree relative with pulmonary fibrosis/idiopathic interstitial pneumonia in the family, and without crackles on lung auscultation? Screening can include pulmonary function testing and/or CT imaging	No, screening is not indicated	40%
	Yes	45%
	Unsure	14%
[If Yes or Unsure] Which test(s) would you recommend?	Only spirometry (with no measurement of lung volumes or diffusion capacity), and no HRCT	0%
	Full pulmonary function tests (spirometry plus measurement of lung volumes and diffusion capacity), but no HRCT	32%
	HRCT alone	11%
	Only spirometry (with no measurement of lung volumes or diffusion capacity) plus HRCT	0%
	Full pulmonary function tests (spirometry plus measurement of lung volumes and diffusion capacity), plus HRCT	58%

[a]Demographics- and free text-related questions are not shown.
CT = computed tomography; ILD = interstitial lung disease; S-modifier = potentially significant incidental finding.

Online supplements are not copyedited prior to posting and the author(s) take full responsibility for the accuracy of all data.
